# Trailblazer in Medicine: The Inspirational Journey of Dr. Anandibai Gopalrao Joshee

**DOI:** 10.7759/cureus.67934

**Published:** 2024-08-27

**Authors:** Krishna Sailaja Sattiraju, NG Nihal, Venkata Subbarao Ayyagari, Kasireddy Sravanthi, Shriraj Katakdhond

**Affiliations:** 1 Obstetrics and Gynaecology, Dr. D. Y. Patil Medical College, Hospital & Research Centre, Dr. D. Y. Patil Vidyapeeth (Deemed to be University), Pune, IND; 2 Psychiatry, Gayatri Vidya Parishad Institute of Health Care & Medical Technology, Visakhapatnam, IND; 3 Pediatrics, Dr. Pinnamaneni Siddhartha Institute of Medical Sciences and Research Foundation, Vijayawada, IND; 4 Pediatrics, Dr. D. Y. Patil Medical College, Hospital & Research Centre, Dr. D. Y. Patil Vidyapeeth (Deemed to be University), Pune, IND

**Keywords:** anandibai gopalrao joshee, biographies, historical vignette, social reform, pioneering women, women's rights, female physicians, medical education, women's healthcare, indian medicine

## Abstract

Dr. Anandibai Joshee, emerging from a patriarchal society in Maharashtra, India, stands as a trailblazer in the annals of medical history, not only as the first Indian woman to earn a degree in Western medicine but also as a fervent advocate for women’s rights. This paper delves into her life, exploring her contributions to the medical field, her advocacy for women's education, and the cultural impact she left on both India and the international community. Her achievements are examined in the context of the medical and socio-cultural milieu of 19th-century India and today, providing a detailed account of her legacy and recognition.

## Introduction and background

**Figure 1:**
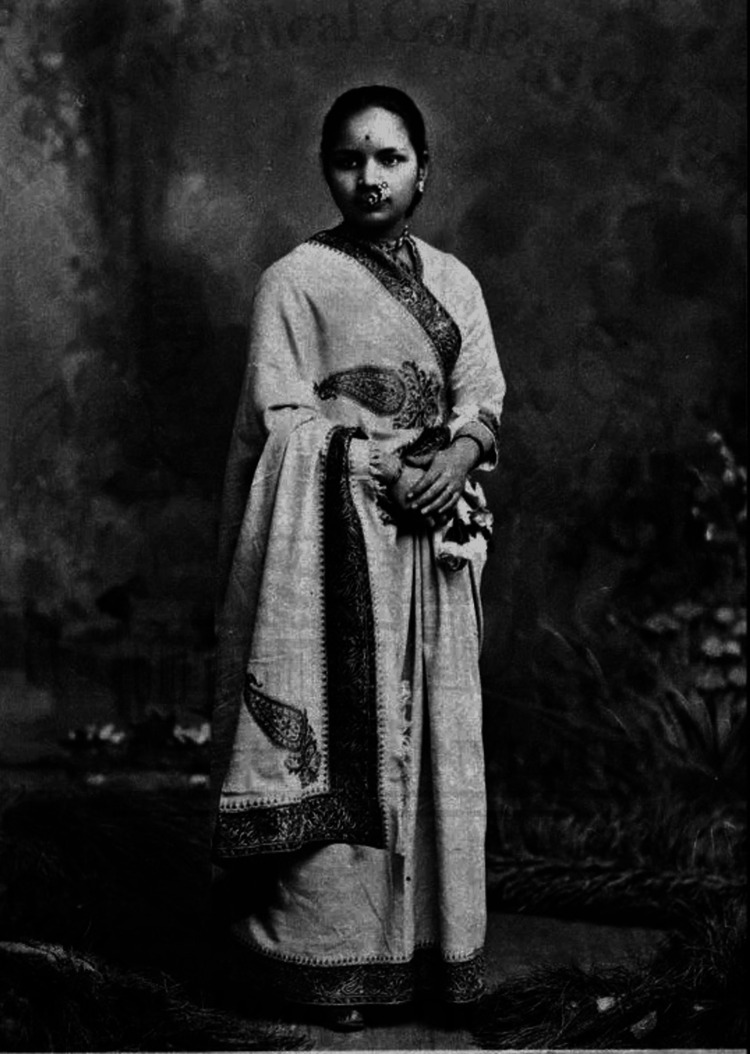
Dr. Anandibai Joshee Source: Wikimedia Commons [3].

Anandibai Gopalrao Joshee (born Yamuna Joshi) (Figure 1) [1] grew up in a conservative Marathi-speaking Chitpavan Brahmin family as the fifth child with eight other siblings in Kalyan, Maharashtra [2]. At the age of nine (the norm at the time), she was married off to Gopalrao Joshee, a widower and a progressive thinker who championed her education despite being 20 years older than her [3]. Gopalrao's encouragement played an instrumental role in Anandibai's pursuit of higher education and a career in medicine, a rare aspiration for women in 19th-century India since it was a field then dominated by men and largely inaccessible to women in India [4,5].

In the 1800s, it was exceptionally rare for husbands to prioritize their wives' education; however, Gopalrao Joshee was deeply committed to Anandibai's academic advancement. His fervent dedication was such that he reacted vehemently when he found her engaging in traditional domestic activities, a stark contrast to the typical gender dynamics of the era.

Gopalrao made several attempts to enroll her in missionary schools in Maharashtra but failed, and they eventually relocated to Calcutta (now Kolkata, India). She picked up reading and speaking English and Sanskrit there, which laid the foundation for her further studies.

## Review

Pursuit of medical education

Anandibai's determination to study medicine was partly fuelled by an immense personal tragedy. At the tender age of 14, she gave birth to a son, who died merely 10 days after birth due to lack of access to medical care and societal taboos in seeking treatment from Western medicine physicians. This incident profoundly impacted her, motivating her to pursue a career in medicine [6].

Her husband wrote to several American missionaries, seeking support for Anandibai's education. In 1880, a renowned missionary in America, Royal Wilder, received a letter from Gopalrao expressing his wife's desire to find out about a suitable position for herself in the States. The correspondence of which he happened to publish in his Princeton Missionary Review [7]. One resident of New Jersey, Theodicia Carpenter, read this very post casually while waiting at an appointment with the dentist. She responded to this post positively, offering to host Anandibai in the United States. These two women would go on to develop a deep familial bond of sorts and come to be each other’s “Aunt-Niece” [3,8].

Despite facing societal backlash and health challenges, Anandibai traveled to America in 1883, enrolling in the Woman’s Medical College of Pennsylvania (WMCP) (now part of the Drexel University College of Medicine), one of the few institutions at the time that accepted female students and only the second to do so [9,10].

Anandibai delivered a speech at the Serampore College Hall in 1883 before leaving for America, elucidating her decision to travel to the United States to pursue a medical degree. During her speech, she detailed the persecution she and her husband endured in the Hindu community, and despite this, she pledged to not convert and continue to fight. She underscored the urgent need for more female physicians in India, highlighting that “Hindu” women would be better suited to provide medical care for other “Hindu” women [9,11].

Medical education and achievements

In 1886, Anandibai graduated with a degree in medicine (MD), making her one of the first Indian women to do so. Her thesis, "Obstetrics among the Aryan Hindoos," was a pioneering work that provided insight into the practices of childbirth and the role of midwives in India. The thesis highlighted the need for medical reforms and the inclusion of women in the healthcare system to improve maternal and child health outcomes [6]. Queen Victoria, who was also the Empress of India at the time, congratulated WMCP on her subject’s success when she graduated [8,9].

Death

Anandibai's life was tragically cut short at the age of 21, on 26 February 1887, due to tuberculosis, in Pune. She had been tired and weak for years before she passed away. She received medicine from America, but it had no effect, so she continued to study it till the day she passed away [6].

**Figure 2:**
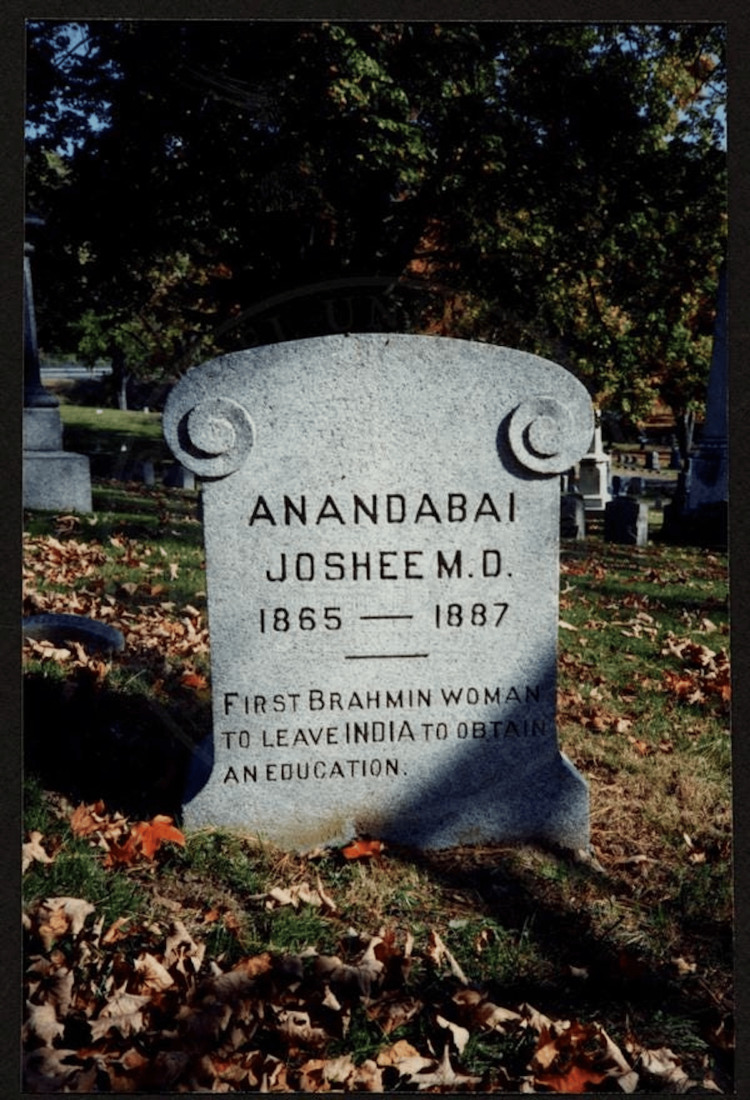
Resting place of Dr. Anandibai’s ashes at the Poughkeepsie Rural Cemetery in New York Source: Drexel University Archives [12].

Her ashes were sent to the United States, to Theodicia Carpenter, and are kept in the Poughkeepsie Rural Cemetery in New York, with the inscription reading, “First Brahmin woman to leave India to obtain an education,” as shown in Figure 2 [12].

Contributions to medicine

Anandibai's journey to becoming a physician inspired many Indian women to pursue medical careers. Her success demonstrated that women could excel in the medical field, challenging the prevailing gender norms and opening doors for future generations of female doctors in India [11]. She was a pioneer in female education in medicine.

Anandibai was a strong advocate for women's health, particularly maternal and child health. Her thesis and subsequent work emphasized the importance of proper medical care during childbirth, which was often neglected in traditional practices, driven by her tragic personal loss. By highlighting these issues, she laid the groundwork for improvements in maternal healthcare in India [11].

Upon her return to India, Anandibai aimed to integrate Western medical practices with traditional Indian healthcare. She believed in the benefits of modern medicine and worked toward establishing hospitals and clinics that could provide comprehensive care, particularly to women who were often denied medical attention due to cultural and social barriers [8]. Anandibai's efforts to reclaim ancient Indian medical knowledge, as well as her subsequent advocacy for its inclusion in modern medical discourse, positioned her as a precursor to contemporary integrative medicine [8].

Anandibai's achievements served as a beacon of inspiration for countless women in India. Her story was widely covered in Indian newspapers and magazines, sparking a movement toward female education and empowerment. Her perseverance and success story encouraged many families to educate their daughters and support their aspirations in various professional fields, including medicine.

Her contributions had a lasting impact on medical reforms in India. She highlighted the critical need for female physicians in the country, particularly to address the healthcare needs of women and children. In her 1883 speech, she said, “I volunteer to qualify myself as one.” Her advocacy paved the way for subsequent reforms that aimed to improve medical education and healthcare services in India [9,11].

Dr. Joshee's achievements did not go unnoticed. In 1886, she was appointed the physician-in-charge of the female ward of the Albert Edward Hospital in Kolhapur, India, marking the first time an Indian woman held such a position [13]. Her pioneering work earned her recognition both in India and abroad, inspiring subsequent generations of women to pursue careers in medicine and other professional fields.

Posthumous recognition

Anandibai Joshee's contributions have been recognized posthumously in various ways. The Government of India issued a commemorative stamp in her honor in 2016, recognizing her contributions to medicine and women's education [14]. Several hospitals and medical institutions in India are named after her, serving as a testament to her enduring legacy in the field of medicine.

Biographies and Documentaries

Numerous biographies, articles, and documentaries have been produced, chronicling her life and achievements. Notably, Anandi was the subject of a brief biography written in 1888 by Caroline Healey Dall, an early American feminist, and a Marathi biography written in 1912 by Kashibai Kanitkar. A fictionalized account of her life was written by S.J. Joshi and released in 1992 by *Stree*. Joshi's Marathi novel *Anandi Gopal* was translated into English by Asha Damle, and more recently, *A Fragmented Feminism* was published posthumously by Meera Kosambi in 2020. These works continue to inspire and educate people about her pioneering contributions.

## Conclusions

Dr. Anandibai Joshee's contributions to the medical field were profound and far-reaching. Her determination to overcome societal and personal obstacles to achieve her goal of becoming a physician was a pioneering effort that inspired many. Through her advocacy for women's health, promotion of medical education for women, and efforts to integrate Western medical practices with traditional Indian healthcare, she played a crucial role in shaping the future of medicine in India. Her legacy continues to inspire and empower women in the medical field, ensuring that her contributions are remembered and celebrated for generations to come.
